# Clinical relevance of loneliness in the treatment of depression: study protocol for a naturalistic prospective longitudinal study

**DOI:** 10.3389/fpsyt.2026.1827062

**Published:** 2026-07-24

**Authors:** Franziska Sonnauer, Franca L. Fries, Maksym Druzenko, Ulrich W. Kastner, Christiane Mühle, Johannes Kornhuber

**Affiliations:** 1 Department of Psychiatry and Psychotherapy, Friedrich-Alexander-Universität Erlangen-Nürnberg (FAU), Universitätsklinikum Erlangen, Erlangen, Germany; 2 Department of Psychiatry, Addiction, Psychotherapy and Psychosomatic Medicine, Bezirkskliniken Mittelfranken, Erlangen, Germany

**Keywords:** biomarker, clinical sample, depression, loneliness, prognostic factor

## Abstract

**Background:**

Loneliness and depression are widespread and severely debilitating health conditions. Notably, loneliness and depression are closely intertwined, with individuals suffering from depression being particularly vulnerable to loneliness and vice versa. However, little is known regarding the clinical significance of loneliness in the treatment of depression and the biopsychosocial mechanisms underlying this association.

**Methods:**

This protocol presents a naturalistic longitudinal study on the clinical significance of loneliness in inpatient and post-inpatient treatment outcomes of adults with depression. The design includes three main assessment points with comprehensive diagnostics (including clinical interviews, questionnaires, and optional biospecimen collection for biomarker and microbiome analyses), as well as interim assessments and the integration of routine clinical data. The primary outcomes include depressive symptom severity and perceived loneliness across the course of treatment and follow-up. Secondary outcomes and covariates are analyzed to identify other clinically relevant indicators, such as life satisfaction and suicidality, and to better understand the biopsychosocial interplay between loneliness and depression. Descriptive and inferential statistical analyses, including (generalized) linear mixed model analyses and mixed analyses of variance (ANOVA), will be conducted following both hypothesis-driven and exploratory approaches.

**Discussion:**

By employing a clinical sample and longitudinal design, this study advances the understanding of the role of loneliness in the treatment and maintenance of depression. In addition to evaluating primary outcomes, analyses of secondary outcomes can provide information on shared and distinct biopsychosocial pathways, thereby identifying potential correlates of mental health outcomes that may inform future research. Limitations include the possible inflation of Type I errors in exploratory analyses and potential biases such as selection bias and cognitive distortions in self-reported data. Nevertheless, this study can provide an important foundation for subsequent clinical trials and translational research. This study was prospectively registered at ClinicalTrials.gov on 31^st^ December 2025 before patient enrollment (Identifier: NCT07333027, Study Details | NCT07333027 | Lonely in Depression | ClinicalTrials.gov).

**Study Protocol Registration:**

https://clinicaltrials.gov/study/NCT07333027?cond=NCT07333027&viewType=Card&rank=1, identifier NCT07333027.

## 1 Introduction

Loneliness can be considered among the most pressing health challenges affecting humans at present. The latest findings of the World Health Organization (WHO) indicate that loneliness affects one in six individuals and that, on average, around 100 people worldwide die every hour due to loneliness-related causes (1). Loneliness refers to the subjective and aversive experience of being alone. The frustration of the need for belonging (2) may exhibit a functional characteristic in the short term (3); however, in the long term, loneliness can lead to destructive emotional-affective (4), metabolic, and immunological processes (5). In particular, chronic loneliness is associated with higher risks of mental (3, 6, 7), cardiovascular (8) and neurodegenerative (9–12) diseases. The prevalence of loneliness in the general population is estimated at 11.1% in Western European countries and has been observed to be even higher in lower-income countries (1, 13).

Loneliness coincides with depression in the general population. A study focused on the general population of older persons in England revealed that, among people with severe loneliness, 3.3% of these individuals (vs. 1.1% of individuals with low/moderate loneliness) demonstrated clinically relevant depression, and 45.2% (vs. 13% of individuals with low/moderate loneliness) exhibited measurable depressive symptoms (14). Another representative population study conducted in Germany revealed that among individuals who reported depression in a self-assessment using the Patient Health Questionnaire, 52.6% reported pronounced loneliness, 30.5% reported moderate loneliness, 19.3% reported minimal loneliness, and only 5.2% reported no loneliness at all (15). Compared with other health consequences that have been investigated, the associations between loneliness and negative health outcomes are most apparent with respect to mental health (3). In conjunction with another meta-analysis conducted by Erzen and Cikrikci (16), the authors reported moderate effects of loneliness on depression. Suicidal tendencies and loneliness are also significantly related (3, 17). The relationship between depression and experiences of loneliness is likely to be bidirectional. However, in non-clinical study populations, longitudinal studies have suggested evidence of a relatively stronger predictive influence of loneliness on depression (18, 19). To our knowledge, there is currently no reliable data regarding the prevalence and clinical relevance of loneliness among patients with clinical depression.

The pathophysiological pathways underlying the adverse health effects of loneliness remain incompletely understood. It is very likely that the best-described pathophysiological mechanism through which loneliness is linked to health involves the chronic overactivation of the hypothalamic-pituitary–adrenal axis (HPA axis) triggered by psychosocial stress. This activation leads to increased glucocorticoid release and resistance; subsequently, numerous consequences are induced, including mitochondrial dysfunction and inflammatory dysregulation, hyperglycemia, and increased vascular resistance (20). At the neurotrophic level, findings suggest that the interaction of loneliness and brain-derived neurotrophic factor (BDNF) is crucial for neuroplasticity and problem-focused coping (23, 24). While cortisol is one of several factors involved in suppressing BDNF expression (25) opposing mechanisms that upregulate BDNF expression, such as physical activity, are also well documented (26). Moreover, loneliness is bidirectionally associated with sleep disturbances, including daytime dysfunction such as fatigue, low energy, and sleepiness (21) and is related to sleep fragmentation (22). Additional mechanisms, such as poorer health behaviors (e.g., reduced physical activity), affective and neuronal dysregulation (involving the cognitive control model) (4, 27, 28), and fewer compensation mechanisms such as a lack of social support (known as the buffer hypothesis) culminate in a vicious cycle (20). Importantly, much of the biological evidence remains correlational and does not permit causal inferences.

Social support mediates the unfavorable interaction between loneliness and depression (29) and, according to prospective studies, is a well-documented protective factor against depression (30–32). On a neuroendocrine pathway social support appears to buffer HPA axis dysregulation (33, 34). In addition to these protective factors, the risk factors for loneliness are closely related to the risk factors for depressive disorders. Risk factors include both individual factors (such as living alone, few social contacts, low socioeconomic status, personality traits, and pre-existing conditions) and the social context, wherein people who are surrounded by lonely individuals and people situated at the margins of social network structures are particularly at risk (35, 36). Therefore, these mechanisms likely interact dynamically and form a biopsychosocial feedback system in which psychosocial stress, biological dysregulation, and behavioral factors reinforce each other. The association between loneliness and depression, as well as general health, likely arises from this complex interaction.

The pathways between loneliness and health have only been partially investigated to date, predominantly using samples from the general population. Initial longitudinal studies have analyzed the interplay between loneliness, depression, and metabolic outcomes. Via the use of harmonized cohort data obtained from longitudinal trials in England, Czechia, and Finland, the authors compared a healthy control group (CG), a group of individuals with depression but no loneliness (DEP), and a group of individuals with both loneliness and depression (DEP+LONE) (37). The measured metabolic markers included the blood sugar level, triglyceride level, and cholesterol level, as well as blood pressure and abdominal circumference. Women in the DEP+LONE group demonstrated significantly more metabolic abnormalities at follow-up than women in the DEP and CG groups (37). This association was not observed for men; in particular, significantly more abnormalities were observed in the DEP group than in the CG or DEP+LONE group, although multimorbidity (measured as the number of comorbid conditions, such as diabetes or respiratory diseases) was most pronounced in the DEP+LONE group in both sexes (37). It remains unclear if these metabolic outcomes are results of poorer health habits or shaped by HPA axis dysregulation or inflammatory processes.

Furthermore, exploratory studies are focusing on investigating the role of the microbiota-gut-brain axis as another multifactorial regulatory system within the context of loneliness. Nguyen and colleagues (38) have found evidence that loneliness in the general population is associated with reduced gut microbiome diversity (also known as alpha diversity), whereas greater microbial diversity is typically considered to be beneficial for health. Moreover, findings on the microbiota-gut-brain axis and depression have been increasingly investigated (39–41). The review of Rosell-Cardona and colleagues (42) provides an overview of changes in the microbiota-gut-brain axis in people with depression compared to healthy controls and points to an increased prevalence of pro-inflammatory bacteria (dysbiosis). While initial findings hint that the composition of the microbiome may have predictive value in forecasting treatment response to antidepressants (43–46), the exact mechanisms remain inadequately understood (43). The reproducible conduct and interpretation of human studies on the microbiota-gut-brain axis are complicated by various lifestyle influences, particularly diet, by medication (antidepressants, antibiotics), and additional interindividual variation arising from, for example, comorbidities. In animal models (rats, mice), established paradigms (e.g., sub-chronic social defeat stress) or fecal transplants are used to investigate depressive behavior and its bidirectional relationship with the microbiota-gut-brain axis (41, 42). As a result, some mechanisms have already been described in greater detail, including the role of tryptophan metabolism in the gut or the protective regulatory effects of short-chain fatty acids on neuroplasticity and blood-brain barrier integrity (42, 47). However — as the differences between the efficacy of probiotics in animal models and human trials already suggest — the direct transferability of these findings to human remains limited, with effects of considerably smaller magnitude observed in human studies. In summary, the microbiota-gut-brain axis interacts bidirectionally with other relevant pathways of depression. This encompasses behavioral [e.g., diet, exercise (48)], neurotrophic, immunological, and endocrine mechanisms.

Although more intensive research is needed to precisely describe the complex relationships between loneliness, pathophysiological processes, and health outcomes, the literature highlights the relevance of loneliness for mental health and overall health. In contrast to the complex effects that loneliness entails, psychometric assessments are relatively straightforward via established survey instruments such as the 3-Item UCLA Loneliness Scale (49, 50) or the 6-Item De Jong Gierveld short scales for emotional and social loneliness (51, 52). However, the literature demonstrates conflicting results regarding the cutoff values chosen to differentiate between existing and nonexistent loneliness. For example, 3-Item UCLA Loneliness Scale total scores range from 3 to 9, and some authors have described individuals with a score of ≥ 4 as ‘lonely’ (53), whereas others have only assumed existing loneliness according to scores of ≥ 6 (13, 14). The authors of the 6-Item De Jong Gierveld short scale specified cutoff values (0–1: not lonely; 2–4: moderate loneliness; and 5–6: severe loneliness); however, for this purpose, a dichotomization of the previously existing three- or five-point response scale is performed, resulting in a total score of 0–6 points (54). The measurement of loneliness demonstrates one additional methodological limitation in that the mentioned instruments do not indicate when loneliness can be considered a ‘chronic’ condition. However, this scenario would be interesting to assess, in that experiences of loneliness exhibit lifetime consistency that is comparable to that of personality traits (known as ‘trait-like features’) (55). This correspondingly indicates that certain people feel lonelier than others throughout their entire lives. The findings from twin and adoption studies on gene-environment interactions have supported this proposition, with a heritability rate of 37–55% being reported (56), supporting the notion that loneliness results from the interaction of individual predispositions and environmental influences. Regarding the treatment of depression and the mainly episodic course of the illness, it seems highly important for patients with depression to be able to better distinguish between loneliness as a *trait-like* feature or as a *state* condition during a depressive episode.

In summary, with respect to the interplay between loneliness and depression, people who suffer from depression are at an increased risk of experiencing loneliness, and loneliness correspondingly represents a predictor of depression. However, the clinical relevance of loneliness in treating depression remains largely unexplored.

Several factors contribute to this research gap: first, scientific literature on the interplay of loneliness and depression refers almost exclusively to a non-clinical population. The most investigated subpopulation involves older adults. The frequently used measures (including the 3-Item UCLA Loneliness Scale and the 6-Item De-Jong Gierveld Loneliness Scale) have not been subsequently evaluated in a clinical population, thereby indicating that there is no verified cutoff point for quantifying clinically relevant experiences of loneliness. Second, the primary analysis design is predominantly cross-sectional or refers to longitudinal observational cohort studies; therefore, it does not allow for conclusions to be obtained regarding individuals’ courses of loneliness and depression. Third, although both loneliness and depression are uncontroversially shaped by biopsychosocial influences, little is known about the shared and non-shared variance of these associations. To understand the interplay between loneliness and depression, it is necessary to further investigate the potential shared mechanisms, including biological mechanisms (such as hormonal activation of the HPA axis or health behaviors) and psychosocial constructs (such as social support or social conditions such as marital status and social network size).

The main objective of the present study is to examine the clinical relevance of the interaction between loneliness and depression in a naturalistic inpatient and aftercare treatment setting. More specifically, the aim of the study is to explore whether there is a significant association between experiences of loneliness and the severity of depressive symptoms over time in a cohort of inpatients. The secondary objectives of the study include an exploratory analysis of biopsychosocial factors and mechanisms related to loneliness and depression. **Supplementary Figure 1** in Section 1 of the **Supplementary Material** schematically integrates these elements into a *hypothesized*, literature-derived framework. Within this framework, loneliness and related psychosocial characteristics are positioned as the upstream domain, depressive symptom severity and other clinical outcomes as the downstream domain, and neuroendocrine, inflammatory, neurotrophic, microbiota-gut-brain, and behavioral processes as intermediate mediating mechanisms, with a reciprocal link between loneliness and depression. The association between loneliness and the course of depressive symptom severity — assessed with validated instruments at both recruitment sites — is central to the confirmatory hypotheses, whereas the biological mediating mechanisms are addressed only in an exploratory, hypothesis-generating manner within the biomarker/microbiome subsample. This framework is conceptual: as the study is non-interventional, the planned analyses estimate associations consistent with the model and do not test the direction of effects or establish causation.

The primary hypothesis states that loneliness (3-Item UCLA Loneliness Scale) at admission, the change in loneliness from admission to discharge, and their interaction (admission x change in loneliness) will significantly predict depressive symptom severity, assessed using the Montgomery–Åsberg Depression Rating Scale (MADRS), in a linear mixed-effects model, tested using an omnibus likelihood ratio test (*α* = .05). As depression is our main outcome variable, we will also address this hypothesis for self-report data, measured with the Patient Health Questionnaire (PHQ-9). The loneliness scores of the 6-Item De Jong Gierveld Loneliness Scale will serve for sensitivity analysis to examine the measurement robustness and construct validity. A more detailed but exploratory analysis of the fixed and random effects and time-varying loneliness and depression within the inpatient and follow-up treatment period will be explored with linear mixed model analysis to 1) examine whether loneliness has a role in the inpatient therapy effect on depression symptom severity and its maintenance after discharge, 2) investigate the clinical relevance of chronic loneliness, and 3) describe the bidirectional relationship between loneliness and depression at all measures. To evaluate the prognostic value of baseline loneliness (3-Item UCLA Loneliness Scale) to predict response at discharge (≥ 50% reduction in MADRS) and the prognostic utility of loneliness at discharge to predict relapse (≥ 10 MADRS points more from discharge to follow-up or ≥ 22 MADRS points at follow-up), receiver operating characteristics (ROC) will be determined and reported in an exploratory fashion. Along with the main objective to investigate the clinical relevance of loneliness and depression, we will test several exploratory hypotheses. Since the duration of inpatient treatment has implications for the allocation of healthcare resources, we will test whether loneliness at admission and change in loneliness from admission to discharge are significant predictors of inpatient length of stay after adjusting for covariates. The secondary objective of the study is to identify the biopsychosocial processes that shape the interaction between loneliness and clinical depression. To this end, statistical analyses of additional clinically relevant outcomes (such as quality of life and suicidality) as well as interactional effects of potentially shared psychosocial mechanisms (including social support, social network, comorbidities, general health and physical activity) will be conducted. Exploratory analysis of blood, saliva and stool markers including metabolic, hormonal, immunological, and neurotrophic factors as well as microbiome diversity will deepen the understanding of pathophysiological processes. We hypothesize that more depressed and more lonely people will have the most unfavorable metabolic profile in blood markers (Low-Density-Lipoprotein, HbA1c), higher levels of inflammation or cellular stress (sphingolipid metabolism) and less neuroplasticity (BDNF). In terms of saliva pattern markers, we assume a higher stress response (cortisol, α-amylase activity) in individuals with more severe depression and loneliness. For stool markers, it is hypothesized that more depressed and lonely people have a less diverse stool microbiome (alpha-diversity). We hypothesize the changes in biomarker profile from baseline to discharge from clinic and baseline to follow-up to be associated with changes in depression severity and changes in loneliness experience.

## 2 Methods and analysis

### 2.1 Study design and setting

To examine the clinical relevance of the interplay between loneliness and depression, a prospective, noninterventional longitudinal design will be adopted. The study is being conducted at the University Hospital and the Community Hospital in Erlangen, Germany. Patients admitted to the hospital with depressive symptoms are eligible for screening according to the inclusion and exclusion criteria. All study procedures — except for the collection of biosamples, which is limited to the University Hospital — will be performed at both centers. Three main measurement points (T0, T1, and T2) and brief interim measurements conducted at two-week intervals (t0.1 to t0.X and t1.1–t1.5) will be utilized. After screening and clinical interviews, participants will complete the baseline measurements (T0). Thereafter, each participant will adhere to an individual period corresponding to the duration of that specific patient’s inpatient stay at the clinic until the second measurement is obtained (T1). The follow-up examination takes place three months after T1 (see **Table 1** for measures and participant timelines and **Supplementary Figure 2** in Section 2 within the **Supplementary Material** for the study flow chart). The SPIRIT table was adapted to the non-randomized study design.

**Table 1:** Adapted SPIRIT schedule.

		Study period				
TIMEPOINT and study procedures	Enrollment	Baseline	Length of inpatient treatment (minimum 7 days)	Discharge	Duration of 3 months after discharge	Follow-up
TIMEPOINT	−t_i_ to 0	T0 + 0 to 7 days	t_0.1_, t_0.2_ etc. t_0.X_	T1 ± 7 days	t_1.1_ to t_1.5_	T2 ± 7 days
ENROLLMENT:						
Eligibility screening	X					
Informed consent	X					
Clinical interview (M.I.N.I. screen and M.I.N.I.)	X					
ASSESSMENTS:						
Outcomes						
Clinical interview						
*Depression – MADRS*		X		X		X
Questionnaires						
*Depression – PHQ-9*		X	X	X	X	X
*Loneliness – 3-Item UCLA*		X	X	X	X	X
*Loneliness – 6-Item DJGLS*		X		X		X
*State vs. trait – 1 Item*		X		X		X
*Social support – F-SOZU-14*		X		X		X
*Social Network Index – adapted SNI*		X		X		X
*Health status – SF-12*		X		X		X
*Quality of life – WHO-5*		X		X		X
*Social Anxiety – SPIN*		X		X		X
*Somatic symptom burden – PHQ-13*		X		X		X
Biosampling^1^						
*Blood*		X		X		X
*Saliva*		X		X		X
*Stool*		X		X		X
Other variables						
Self-report questionnaire						
*Personal information (e.g., height, weight, and treatment course)*		X		X		X
*Lifestyle information (e.g., substance use, physical activity, and diet)*		X		X		X
Routine clinical data						
*Setting*		X		X		X
*Comorbidity*		X		X		X
*Inpatient treatment duration*		X		X		X
*Medication*		X		X		X

M.I.N.I. Screen, Mini International Neuropsychiatric Interview Screening Instrument; M.I.N.I., Mini International Neuropsychiatric Interview; MADRS, Montgomery and Åsberg Depression Rating Scale; PHQ-9, 9-Item Patient Health Questionnaire; 3-Item UCLA, 3-Item UCLA Loneliness Scale; 6-Item DJGLS, 6-Item De-Jong Gierveld van Tilburg Loneliness Scale; F-SOZU-14, Fragebogen Zur Sozialen Unterstützung, Kurzform; adapted SNI, adapted version of Social Network Indices; SF-12, Short Form 12 Health Survey; WHO-5, World Health Organization-Five Well-Being Index; SPIN, Social Phobia Inventory; PHQ-13, 13-Item Somatic Symptom Severity Scale of the Patient Health Questionnaire.

^1^ Footnote to **Table 1**: Biosampling (blood, saliva, stool) is performed at the University Hospital site only.

### 2.2 Inclusion and exclusion criteria

To be included in this study, individuals must meet the following inclusion criteria: (A) inpatient elective or emergency admission to one of the two psychiatric clinics; (B) age over 18 years; (C) sufficient understanding of spoken and written German; (D) primary diagnosis of depression according to ICD-10 (F32 or F33), as diagnosed by a physician or clinical psychologist in conjunction with the M.I.N.I. Screen [Mini-International Neuropsychiatric Interview Screen (57)] and M.I.N.I. [Mini-International Neuropsychiatric Interview (58–60)] diagnostic tools; and (E) informed voluntary consent. Diagnoses are coded according to the International Classification of Diseases (ICD-10-GM) applicable in Germany, both for psychiatric disorders and for non-psychiatric comorbidities. The M.I.N.I., a structured clinical interview, is based on both the Diagnostic and Statistical Manual of Mental Disorders (DSM-V) and the ICD-10-GM. This additional instrument is intended to supplement the diagnostic process, increase diagnostic certainty, and better characterize suicidality and psychiatric comorbidities, with our focus particularly on anxiety disorders. The following exclusion criteria were applied: (a) a current lack of capacity to provide consent (e.g., pronounced psychotic symptoms, stuporous depressive syndrome, and thought constriction to suicidality); (b) previous participation in the study; (c) pregnancy or breastfeeding; and (d) an inpatient stay of less than 7 days, even if the patients initially met the inclusion criteria (post-enrollment exclusion).

### 2.3 Sample size consideration

Data from previous longitudinal and meta-analytic evidence examining the association between loneliness and depressive symptoms is available for non-clinical populations without any treatment procedure. While cross-sectional data shows correlations of *r* ≈ .50 between loneliness and depression (16, 19), effect sizes are noticeably smaller for longer measuring intervals. For instance, Cacioppo and colleagues (19) reported standardized regression coefficients of *β* = 0.18 in predicting changes in depressive symptoms due to loneliness over one-year and no significant effect of depressive symptoms on loneliness. The 2-year cross-lagged analysis of non-clinical data of the Survey of Health, Ageing and Retirement in Europe (SHARE) however showed that loneliness only in men was a significant predictor of depression (two-year-lagged effect *β* = 0.07), The other way around depression was a small but significant predictor of loneliness in both sexes (*β* = 0.06 for the entire sample, *β* = 0.35 for women, *β* = 0.09 for men) (61). Despite this, these non-clinical references are hardly comparable to the setting of potential associations within and after a multimodal inpatient treatment for depression with an estimated observation period of 4 to 6 months per participant. Given that the effect sizes of the interaction between loneliness and depression over time in a clinical population are currently unknown, the sample size for this study was determined based on statistical plausibility and considerations of practicability and the population generalizability of the obtained results. The theoretical assumptions of our study are that individuals who are more severely lonely at the start of treatment and individuals who experience less reduction in their experience of loneliness during depression inpatient treatment are more likely to have higher levels of depression across baseline, discharge, and follow-up assessments. Statistically the primary hypotheses will be tested using a linear mixed-effects model with depressive symptom severity (MADRS) and loneliness (3-Item UCLA Loneliness Scale) each assessed at admission (T0), discharge (T1), and follow-up (T2). The primary outcome is the severity of depressive symptoms, as assessed using the MADRS at three time points: at admission (T0), at discharge (T1), and at the follow-up visit (T2). The null hypothesis states that neither baseline loneliness (*β*_1_) nor change in loneliness while inpatient treatment (*β*_2_) nor the interaction effect of both (baseline x change) (*β*_3_) will have an association with the change of depression symptom severity (see **Supplementary Material** Section 3 and **Supplementary Figure 3** for the illustration of the primary hypothesis).

As empirical assumptions for the interplay between depression and loneliness in the inpatient and follow-up depression therapy are rare, we decided to set a change of 2 points in the MADRS per 1 SD of loneliness (3-Item UCLA Loneliness Scale) as the minimum meaningful association that shall be detected via the interaction (baseline x change) over the inpatient and post-treatment course. The model tested baseline loneliness and change in loneliness with at least 1.75 points MADRS as we have no empirical assumptions about which association is more significant for this setting, but we have a theoretical assumption that these single associations are less significant than their interaction. An omnibus likelihood ratio test will evaluate these assumptions in a single model, followed by Holm-Bonferroni corrected single tests to evaluate these three assumptions. This step will also evaluate the direction of effects and theoretical plausibility (more loneliness at baseline and less change in loneliness shall predict higher depressive symptom severity). The primary analysis of the linear mixed model includes all three measurement points as a categorial factor. Potential time- effects will serve as covariates in this model and will be reported exploratory with no influence on sample size calculation. Studies examining the clinical course of inpatient depression treatment measured with the MADRS report partly comparable values of MADRS at baseline (≈ 30 points) (62–64) and discharge, though findings remain heterogeneous (65, 66). Model assumptions for this sample size calculation were therefore based on plausible clinical values: T0 = 30, T1 = 16, T2 = 15 MADRS points, with a constant standard deviation of *SD* = 8 across all time points. Between-person differences in depression symptom severity (MADRS) were considered with fixed and random slopes, and ICC was set at .50. Power simulation involved assumptions of approximately 5% missing completely at random (MCAR) and 10% missing at random dropout at T2 (MAR). Sample size estimation was performed with R using the following packages: lme4, lmerTest, MASS and tidyr. The simulation for the overall model yielded a statistical power of ≥ 80% for all three assumptions at *n* = 172 with 15% combined dropout rate (MCAR, MAR), resulting in a total of *n* = 199. The number of simulations was 500 (nsim = 500) at a significance level of *α* = 0.05 for the omnibus likelihood-ratio test, with Holm-Bonferroni corrected single hypothesis tests. As depression can lead to cognitive distortion, correlations between loneliness and depression might be dependent on the measurement point, and given the insufficient clinical validation of the loneliness measures, it remains unclear whether the assumptions of a normal distribution and the assumed level of measurement adequately describe the interplay between depression and loneliness. Additional statistical methods are employed in an explorative and descriptive manner (generalized linear mixed-effects model and mixed analysis of variance). Power-analysis for mixed ANOVA using G*Power Version 3.1.9.7, with categorical groups of ‘high’ and ‘low’ loneliness (median-split) when considering an α error of 0.05, a power of 0.9 (1-β error probability) and a correlation value among repeated measures of 0.5 for the group’s results an estimated sample size of *n* = 36 participants assuming a medium effect size (*f* = 0.25), and *n* = 214 participants assuming a small effect size (*f* = 0.10).

To ensure the external validity of the results, it is important that both severely lonely depressive patients and less lonely depressive patients, as well as different disease trajectories and patient characteristics (e.g., age, sex, and number of comorbidities), can be identified. A sample size of approximately 200 participants is expected to ensure sufficient heterogeneity. This sample size is feasible in practice, since even taking into account an inclusion rate of 50% and a dropout rate of 30% for the primary diagnosis of depression at the University Hospital and the Community Hospital in Erlangen, the recruitment potential significantly exceeds the target number of cases within an estimated period of no more than two years.

### 2.4 Measures

#### 2.4.1 Primary outcomes

To increase the validity of the primary outcome measures and reduce inherent bias for depression, a clinician-rated instrument (Montgomery–Åsberg Depression Rating Scale or MADRS) and a self-report instrument (9-Item Patient Health Questionnaire or PHQ-9) will be employed. However, loneliness is conceptualized as a fundamentally subjective construct. Therefore, we will use the two most established scales (the UCLA Loneliness Scale and De Jong Gierveld van Tilburg Loneliness scales), each in their abbreviated form. To capture the characteristics of the social network as a more objectively accessible domain, an adapted version of the Social Network Indices measure is used within the assessment battery. Furthermore, we administer an additional, newly developed single item intended to capture situational (state) versus enduring (trait) aspects of loneliness. Neither the adapted German Social Network Index nor this single item have been psychometrically validated and therefore cannot be used as established measures. Accordingly, any analyses involving these measures are strictly exploratory, and are not interpreted as validated indicators of objective network size or state versus trait loneliness.

##### 2.4.1.1 Depression

###### 2.4.1.1.1 Montgomery–Åsberg Depression Rating Scale (MADRS)

Core symptoms of depression will be assessed by using the MADRS (67, 68), which is a widely used semi structured clinician-rated interview consisting of 10 items (*apparent sadness, reported sadness, inner tension, sleep, appetite, concentration, lassitude, inability to feel, pessimistic thoughts, and suicidal thoughts*). Each of these items is rated on a scale from 0 to 6, with a sum score ranging from 0 to 60 points. Higher scores indicate greater severity of depressive symptoms, with scores of 31 or above suggesting the presence of severe depressive syndrome (69). The assessment refers to the preceding seven days and is considered to be suitable for repeated measurements (68, 70).

###### 2.4.1.1.2 Depression (self-report): 9-Item Patient Health Questionnaire (PHQ-9)

The PHQ-9 (71, 72) is a self-report measure that is used to assess depressive symptomatology and is frequently used in both clinical and non-clinical populations (73, 74). It comprises nine items reflecting the DSM-IV criteria for major depressive disorder but remains one of the most frequently used self-report measures. The PHQ-9 is rated on a four-point Likert scale (0 = *not at all*, 1 = *on several days*, 2 = *on more than half of the days*, and 3 = *nearly every day*). The sum score ranges from 0 to 27 and is used to evaluate the severity of depressive symptoms. A score of 20 or higher is indicative of severe depressive syndrome. Moreover, scores ≤ 4 denote no or minimal symptoms, scores between 5 and 9 indicate mild symptoms, scores between 10 and 14 indicate moderate symptoms, and scores between 15 and 19 indicate moderately severe depressive symptoms.

##### 2.4.1.2 Loneliness

###### 2.4.1.2.1 3-Item University of California, Los Angeles (UCLA) Loneliness Scale (3-Item UCLA)

The 3-Item version (49) is a shortened form of the original 20-Item UCLA Loneliness Scale (75–77) and demonstrates comparable main psychometric criteria (50) and thus benefits regarding secondary test criteria (economy, acceptability). Loneliness is measured through three items, including (1) *feeling a lack of companionship*, (2) *feeling left out*, and (3) *feeling isolated from others*, which are rated on a three-point Likert scale (1 = *hardly ever/never*, 2 = *some of the time*, and 3 = *often*). The resulting sum score (range: 3–9) reflects the degree of perceived loneliness, with higher scores denoting greater loneliness. Cutoff values vary across different studies, although thresholds above 4 or 6 points are most frequently used to classify individuals as ‘lonely’.

###### 2.4.1.2.2 6-Item De Jong Gierveld van Tilburg Loneliness Scale (6-Item DJGLS)

The 6-Item short version, similar to the original 11-Item scale (51), captures two dimensions of loneliness, so-called social and emotional loneliness, but can also be used as a unidimensional measure of overall loneliness. Again, the shorter version was found to have validity and reliability comparable to the longer version (51). Each of the six statements is rated on a three-point scale (1 = *no*, 2 = *more or less*, and 3 = *yes*). Before the total score is calculated, the items are dichotomized, thus resulting in a sum score ranging from 0 to 6, wherein scores of 0–1 indicate no loneliness, 2–4 indicate moderate loneliness, and 5–6 indicate severe loneliness (51). The measure demonstrates good reliability and sufficient content validity (52).

#### 2.4.2 Secondary outcomes

##### 2.4.2.1 Social support: Fragebogen zur sozialen Unterstützung, Kurzform (F-SozU-14)

Social support is measured with the F-SozU (78), which is a 14-item German short form that demonstrates good reliability (Cronbach’s *α* = 0.80 or higher) and criterion validity. The instrument comprises 14 items assessing instrumental, social, and emotional dimensions of support. Responses are scored on a five-point Likert scale ranging from 0 *(‘does not apply’)* to 4 *(‘applies exactly’)*, with higher total scores indicating higher levels of perceived social support.

##### 2.4.2.2 Social network: adapted social network index (adapted SNI)

The Social Network Indices measure (79–81) reflects the degree of social integration or isolation. In the present study, an adapted and German-translated version was used to capture participants’ social interaction partners during the prior two weeks. The German adaption was developed specifically for the present study and represents a newly compiled version, as no German validated version is available, awaiting formal validation. Via the use of a yes/no response format, participants indicated whether they had personal or telephone contact with each of the following: (1) *spouse or partner*, (2) *own children*, (3) *parents or parent-like figures*, (4) *other relatives (e.g., siblings, uncles, or aunts)*, (5) *friends*, (6) *neighbors*, (7) *colleagues*, (8) *people known through voluntary activities*, (9) *in-laws (parents, sons, or daughters-in-law)*, and (10) *members of their social group (e.g., community, congregation, sports, or music clubs)*. In line with the structure and logic of validated English Social Network Indices, higher total scores indicate greater social connectedness. Because this adapted German version has not been formally validated, its scores may be subject to measurement bias (e.g., altered factor structure, item non-equivalence relative to the original instruments, or ceiling effects). Accordingly, all analyses involving the adapted Social Network Index are exploratory and hypothesis-generating, and its results are interpreted descriptively rather than as validated indicator of social network structure.

##### 2.4.2.3 Health status: short form 12 health survey (SF-12)

The SF-12 (82–84) captures the health-related quality of life and retains 12 items of the longer SF-36 that represent eight dimensions of physical and mental well-being. The two composite scores (including the physical component summary and mental component summary) are standardized (*M* = 50, *SD* = 10), demonstrate acceptable Cronbach’s *α* > 0.80 and have been validated in various countries and populations. Higher values indicate better health.

##### 2.4.2.4 Quality of life: WHO-five well-being index (WHO-5)

The World Health Organization Well-Being Index (WHO-5) (85) is a brief and universally applicable measure of general well-being over the past two weeks. Each of the five items is positively phrased (e.g., *‘I have felt calm and relaxed’*) and rated on a six-point Likert scale (0 = *at no time*, 1 = *some of the time*, 2 = *less than half of the time*, 3 = *more than half of the time*, 4 = *most of the time*, and 5 = *all of the time*). The total score (0–25) is transformed to a scale ranging from 0 to 100, with scores ≤50 indicating diminished well-being.

##### 2.4.2.5 Social anxiety: social phobia inventory (SPIN)

The German version of the Social Phobia Inventory (SPIN) (86, 87) is a 17-item self-report measure designed to assess multiple dimensions of social anxiety, including physiological arousal (e.g., ‘*My heart is racing when I am with other people.’*), fear of negative evaluation (e.g., ‘*I am very afraid of being criticized.*’), and avoidance behavior (e.g., ‘*I avoid activities in which I am the center of attention.*’). Responses are scored on a five-point Likert scale (0 = *not at all* and 4 = *extremely*), thereby reflecting the extent of distress associated with each item. The German version also exhibits good psychometric criteria (Cronbach’s *α* = 0.94; test-retest *r* = 0.88).

##### 2.4.2.6 Somatic symptom burden: 13-item somatic symptom severity scale of the patient health questionnaire (PHQ-13)

Somatic symptoms will be assessed by using the German version of the Patient Health Questionnaire (72, 88). The psychometric validity of the PHQ-13 is comparable to that of the PHQ-15; however, the former measure enhances usability and gender neutrality. Thirteen physical symptoms are scored over the preceding four weeks, including the degree of associated impairment (0 = *not impaired at all*, 1 = *slightly impaired*, and 2 = *severely impaired*).

##### 2.4.2.7 Inpatient treatment duration

The number of days from the beginning of inpatient treatment to the day of discharge from inpatient therapy will be recorded.

#### 2.4.3 Other variables

##### 2.4.3.1 Biosampling

Biological samples (including blood, saliva, and stool samples) are collected at each assessment point (T0, T1, and T2). Since the analysis of biological samples is exploratory and for hypothesis-generating purposes in this study, we strive to reduce method bias variance by restricting biosampling to the university hospital site to minimize preanalytical errors and ensure procedural consistency. Blood sampling is coordinated with routine clinical blood collections. An additional volume of up to 28.2 ml of venous blood is collected (2 × 9 ml EDTA tubes, 1 × 2.7 ml EDTA tubes, and 1 × 7.5 ml serum tubes). Routine laboratory analyses, including analyses of metabolic markers such as Low-Density-Lipoprotein, High-Density-Lipoprotein, and HbA1c, are performed by the Central Laboratory of the University Hospital Erlangen. The remaining samples are centrifuged, processed (red blood cell lysis for lymphocytes), aliquoted, and stored at -80 °C in the certified biobank (DIN EN ISO 20387) of the University Hospital Erlangen, which is a member of the German Biobank Network. To ensure consistency and comparability of the results of the biochemical analyses, the time between blood collection and the start of processing in the certified biobank is less than two hours. Metabolic, immunological, and hormonal parameters, as well as neurotrophic factors (such as BDNF) are subsequently analyzed by using commercially available antibody-based ELISAs and complementary detection techniques. The activity of enzymes of the sphingolipid metabolism, which are involved in cellular signaling pathways and crucial for maintaining cell membrane structure, will be measured with established assays employing fluorescently labeled substrates (89, 90).

Participants collect saliva samples themselves 30 minutes after awakening (indicating the cortisol awakening response) and a maximum of 30 minutes before bedtime to capture the circadian rhythm of HPA axis activity (91, 92). An evening sample is considered to be a practical surrogate measure of basal HPA axis function, whereas a diurnal nadir typically occurs during nocturnal sleep. All of the saliva samples are collected by using Salivette^®^ (93) and stored at minimum -20 °C in the central biobank of the University Hospital Erlangen until further analysis.

Stool samples are also self-collected by participants by using a collection kit involving a collection tube with stool stabilizer and a feces catcher. Stool samples are stored at -80 °C in the central biobank of the University Hospital Erlangen until subsequent 16S rRNA-based microbiome analysis.

##### 2.4.3.2 Suicidality

Suicidality will be measured in clinical interview (Montgomery–Åsberg Depression Rating Scale (MADRS), Item No. 10 out of 10 items, rated 0-6, with higher values representing more suicidality) and self-report (9-Item Patient Health Questionnaire (PHQ-9) (Item No. 9 out of 9, rated 0–3 with higher scores indicating more suicidality). Completed suicides are recorded as serious incidents.

##### 2.4.3.3 Loneliness: 1-Item state vs. trait

Given the absence of an established measure capable of differentiating between enduring (trait) and situational (state) loneliness, this aspect is captured by using a newly developed single item: *‘In looking back upon my life, I would say that I have felt …’*. Participants respond to this item on a four-point scale (1 = *mostly lonely throughout my life*, 2 = *lonely from time to time*, 3 = *only lonely at present*, and 4 = *never lonely*). Responses of 1 are intended to represent trait loneliness; options 2 and 3 intend to represent state loneliness; and option 4 indicates no loneliness. The item developed for the study has not been validated. The psychometric properties are being examined on an exploratory basis, even though this study does not meet the methodological requirements for validating a single-item-scale. Accordingly, results from this item will not be used to draw conclusions about whether a patient’s loneliness is of a state or trait nature.

##### 2.4.3.4 Self-reports on personal and lifestyle information

Structured assessments are conducted to obtain demographic information and potential covariates associated with treatment outcomes and biomarker levels. The data includes personal characteristics (including sex, age, body weight, height, living situation, employment status, and marital status) and clinical history of depression (including familial occurrence, number of previous episodes, suicide attempts, hospital admissions, and current treatment). Lifestyle variables encompassing substance use (such as smoking, alcohol, cannabis, caffeine, and other drugs), physical activity (including aerobic and resistance training), and dietary habits (including health-conscious diets, yogurt consumption, and recent antibiotic use) are surveyed.

##### 2.4.3.5 Routine clinical data

Routine clinical data, including number of therapies participated, treatment setting, medication, and comorbidities, are retrieved from hospital records by the study physicians. Routine clinical data is recorded and classified in a structured manner, for example, medication is structured by substance classes (SRI/SNRI/NaSSA(Mirtazapin)/NDRI (Bupropion)/TZA/MAO-I/SARI (Trazodon)/Agomelatin/other) and treatment strategy (monotherapy/augmented therapy and predefined changes in dosing or substances).

### 2.5 Data collection

Eligibility screening and clinical diagnostic screening, along with interviews, are conducted by study physicians, supported by trained study psychologists. Depression is assessed during a clinical interview (MADRS) by the patient’s treatment team, which also determines when the patient will be discharged from the hospital. All of the self-report assessments are collected by the patients themselves using the REDCap (94, 95) online survey application. Biosampling will be exclusively performed among participants recruited at the University Hospital to ensure methodologically consistent and rapid processing. Routine clinical data are primarily extracted from clinical documentation systems and consolidated prior to analysis (similar to other data sources).

### 2.6 Statistical analysis

To perform the statistical analysis, IBM SPSS Statistics Version 29 (96) and R (97) are used. To address the primary objective of this study, which is to examine the clinical relevance of the interplay between loneliness and depression in an inpatient and post-inpatient treatment setting, a set of statistical approaches is employed. To address the primary hypothesis, a linear mixed model with likelihood ratio test will be conducted to test against the null hypothesis that baseline loneliness, change in loneliness or their interaction have no additional association with the symptom severity in the course of depression (T0, T1, T2). In the primary linear mixed-effects model, depressive symptom severity (MADRS) is modeled across the three main assessment points (T0 = admission, T1 = discharge, T2 = three-month follow-up), with time entered as a categorical factor. Baseline loneliness at T0 (applied to T0, T1, and T2), the change in loneliness from admission to discharge (applied to T1 and T2), and their interaction (baseline × change) are entered as fixed effects, alongside random intercepts and random slopes capturing individual differences in symptom level and course. Because the length of inpatient stay differs between participants, the individual inpatient treatment duration (days from admission to discharge) is included as a time-related covariate rather than assuming a fixed interval between T0 and T1. The biweekly interim assessments are not part of the primary model and are reserved for the exploratory within-/between-person trajectory analyses described below. The primary test is an omnibus likelihood-ratio test of the null hypothesis that baseline loneliness, change in loneliness, and their interaction jointly show no association with the course of depressive symptom severity; only if this omnibus test is significant are the three individual coefficients examined, with Holm–Bonferroni correction. The formal model specification and the complete list of covariates are provided in the **Supplementary Material** (Section 4 and **Supplementary Table S1**). All models will be controlled for time-effects (e.g., length of inpatient treatment duration), measures obtained at the same timepoint (e.g. explorative analysis of measurement specific associations of loneliness and depression), recruitment site, gender and age and will be tested for further covariates such as social anxiety and social support. The primary analysis sample will include all participants who completed T0 and T1 depression severity measures (MADRS) and loneliness measures (3-Item UCLA). For each questionnaire, missing individual data points are imputed using person-mean imputation, provided that no more than 20% of the data is missing. Participants with an inpatient stay shorter than seven days (post-enrollment exclusion criterion) are not part of the primary analysis set, since no discharge assessment (T1) is obtained for them; where baseline (T0) data are available, they are retained in the sensitivity analyses of all available data. The extent and pattern of missing values will be described for all inference statistical analysis. Given that dropout is likely associated not only with observed characteristics (MAR) but also with unobserved post-discharge symptom severity (MNAR), sensitivity analyses will include pattern-mixture models or selection models to evaluate the robustness of primary findings under MNAR assumptions. As a pragmatic benchmark, a worst-case analysis will be performed assuming that all dropouts at T2 experienced no improvement (last observation carried forward) or deterioration (fixed offset of +5 MADRS points).

Sensitivity analysis will be performed including all available participant data (including T0 only post-enrollment exclusion analysis and analysis of cases with missing data at T0/T1), and further covariates and confounding variables. Additional covariates as medication, comorbidities, social network size and therapy participation, among others, are recorded and classified in a structured manner, for example, medication based on substance classes and treatment strategy (monotherapy/augmented therapy and changes in dosing or substances). Loneliness is being exploratory evaluated in terms of its significance and clinical relevance as a potential predictor of the course and change in severity of depression. For a more differentiated analysis, within-person and between-person variation will additionally be distinguished in an exploratory manner — both for depressive symptom severity and its individual change, and for loneliness and its individual change. The biweekly interim assessments of loneliness and depression are incorporated into this exploratory, hypothesis-generating part of the analysis, which is oriented toward potential intervention targets. It should be noted that, at the interim time points, loneliness is available from the 3-Item UCLA Loneliness Scale, whereas depressive symptom severity is available by self-report only. Accordingly, the PHQ-9 is administered at the biweekly assessments — and likewise at T0, T1, and T2 — to capture self-reported depressive symptom severity.

Secondary outcomes (quality of life, suicidality, social support and others) and biosamples will be analyzed exploratively and reported descriptively. To account for multiple testing in exploratory analysis, p-values will be adjusted using the Benjamini-Hochberg false discovery rate (FDR) procedure. To evaluate prognostic sensitivity and specificity of loneliness measures loneliness at the beginning of inpatient treatment and at discharge, cutoff values for loneliness scores (with 3-Item UCLA and 6-Item DJGLS Loneliness Scales being considered separately) are assessed exploratory by using ROC analysis. The severity of depression symptoms serves as reference standard for predicting response, which is defined as ≥ 50% reduction in MADRS scores from baseline (T0) to discharge (T1), and depression symptom severity measured with MADRS at T2 serves as reference standard for predicting relapse (defined as one or both of the following conditions: ≥ 10 MADRS points more from discharge to follow-up or ≥ 22 MADRS points at follow-up measurement), respectively. The exploratory ROC results are strongly sample-dependent. Because the cutoff values are derived from the present sample, their predictive value and transferability are markedly limited; the ROC analysis should therefore be regarded only as a first attempt at course- and risk-stratification, the validity of which would need to be confirmed in independent samples. A detailed descriptive presentation of all the measurement time points and all variables assessed will be performed, as well as an exploratory analysis using (generalized) linear mixed models.

## 3 Discussion

Although loneliness is currently receiving increased attention as a factor that is relevant to public health, and the fact that people with depression can be considered a risk group, there have been no studies to date on the relevance of loneliness to the treatment of depression. Thus, this naturalistic prospective study provides important insights into whether and to what extent loneliness is relevant for the treatment of depression. A variety of self-report instruments, clinical interviews, routine data, and biosampling approaches are employed for this purpose. The multimethod and longitudinal approach reduces measurement method variance; however, the predominantly exploratory consideration of secondary outcomes and biosamples also carries the risk of determining invalid conclusions, which need to be controlled by Benjamini-Hochberg FDR, covariates or be additionally tested in a subsample and validated by another subsample (known as split-sample validation).

Nevertheless, certain other limitations need to be considered. First, there are methodological difficulties involved in measuring loneliness. Loneliness is a subjective construct; in the context of depression, cognitive and affective distortions can occur, thus indicating that there is a risk that people with severe depression will demonstrate a more negative assessment. Another methodological limitation is that the measures used to assess the constructs of loneliness and depression may lack discriminant validity. An exploratory factor analysis could highlight such overlaps in a salient manner. In addition, the adapted German Social Network Index used in this study has not been formally validated; potential measurement bias cannot be excluded, and analyses based on this instrument are therefore exploratory. Interpretation is further complicated by the differing dimensionality of the instruments used to measure the construct of loneliness: whereas the 3-Item UCLA Loneliness Scale is unidimensional, the 6-Item DJGLS is bidimensional, which may affect how the relationship between loneliness and depression is understood. Although the scales are well-established and used in various countries and cultural contexts, the present sample is probably too small in size to examine cultural influences on the experience of loneliness or the tendency toward socially desirable responses, making biases possible. Altered response behavior may also occur due to repeated assessments (which is known as measurement reactivity) (98). These limitations can only be partially overcome by considering random effects of depression and loneliness and by varying the frequency of loneliness measurements during the study design phase (involving the use of the 3-Item UCLA Loneliness Scale every two weeks and the 6-Item DJGLS at three main measurement points). Self-selection bias may also be a potential limitation, thus indicating that patients with specific levels of loneliness or depression are more likely to participate; moreover, the representativity of the sample in terms of age or sex may be limited. Where available, reasons for non-participation will be reported transparently. Second, several analyses are explicitly exploratory and hypothesis-generating rather than confirmatory. In particular, the ROC-based cutoffs for response and relapse prediction are derived from the present sample and therefore subject to optimism bias; the resulting sensitivity, specificity, and threshold estimates require external validation before any clinical use. Likewise, biomarker and microbiome analyses rest on a site-restricted convenience subsample (biosampling at the University Hospital only) and are interpreted only as preliminary, generalizability-limited findings. This subsample may differ systematically from participants recruited at the community hospital in terms of illness severity, comorbidity profiles, or sociodemographic characteristics, as university hospital populations tend to include more treatment-resistant or complex cases. To mitigate false-positive results across the exploratory analyses, p-values are adjusted using the Benjamini–Hochberg false discovery rate, and key findings are, where feasible, examined by split-sample validation. To evaluate the comparability of both recruitment sites, baseline characteristics will be compared descriptively across centers, and the primary mixed model analyses will include study site as a covariate. Importantly, the primary hypotheses rely exclusively on clinical interview and self-report data (MADRS and 3-Item UCLA; additionally, PHQ-9 and 6-Item DJGLS) that are collected at both sites, ensuring that the main study objectives are testable independently of biomarker availability. Third, despite the longitudinal design of the study, the primary mixed model analysis of this initial non-interventional study on the association of loneliness and depression and their clinical relevance comes with the limitation of reverse causality.

Given that the study design does not permit definitive causal conclusions to be determined about the direction of all fixed and random effects, the findings are still expected to provide valuable insights and relevant implications. In particular, the following insights can be obtained (1): a report on whether patients with different levels of loneliness receive equal benefits from inpatient multimodal depression treatment; (2) insights into changes in loneliness during and after depression treatment; (3) important preliminary findings regarding the shared and distinct biopsychosocial mechanisms underlying loneliness and depression; and (4) the relationship of loneliness and depression on to secondary outcomes, including quality of life and suicidality.

Should the primary hypotheses be confirmed — that is, if baseline loneliness, change in loneliness or their interaction is significantly associated with the level of depressive symptom severity across the treatment course — this may carry implications for clinical practice. First, a brief loneliness screening (requiring less than two minutes using the 3-Item UCLA Loneliness Scale) might be considered for integration into routine psychiatric admission assessments, as it may enable clinicians to identify patients at risk for poorer treatment outcomes early in the treatment process — though randomized controlled studies would be needed to determine whether such screening translates into improved clinical outcomes. Second, the exploratory distinction between more situational and more enduring aspects — examined here only descriptively and without a validated state-trait measure — may, if associated with the course of depressive symptom severity, generate hypotheses for future interventional studies. Such studies could evaluate whether loneliness-focused components (e.g., cognitive restructuring of maladaptive social cognitions or facilitated social re-engagement during the transition from inpatient to outpatient care) add value to multimodal depression treatment. Any inference about modifiability would require an interventional design and lies beyond the scope of the present observational study. Third, the finding that loneliness at discharge predicts relapse would underscore the importance of addressing social connectedness as part of discharge planning and aftercare, a dimension that is currently underrepresented in German psychiatric treatment guidelines.

Several design features strengthen the evidential value of this study. The bicentric recruitment across a university hospital and a community psychiatric hospital enhances ecological validity by capturing a broader spectrum of clinical severity, treatment cultures, and patient populations than a single-site design would permit. The longitudinal design with three main assessment points and biweekly interim measurements allows for the modeling of individual symptom trajectories rather than relying on pre-post comparisons alone. The multimethod approach — combining clinician-rated interviews (MADRS), validated self-report instruments (PHQ-9, 3-Item UCLA Loneliness Scale and 6-Item DJGLS), biological sampling, and routine clinical data — reduces shared method variance and enables triangulation of findings across measurement modalities. Furthermore, the integration of both established loneliness scales alongside a newly developed state-trait item allows for the examination of construct-level questions that cannot be addressed with a single instrument.

Regarding generalizability, the study population comprises adults with a primary diagnosis of depression admitted to inpatient psychiatric care in a German metropolitan area. This setting reflects the structure of the German psychiatric care system, in which inpatient treatment typically involves multimodal therapy programs of several weeks’ duration — a model that differs substantially from shorter inpatient stays common in other healthcare systems (e.g., the United States or the United Kingdom). Consequently, the absolute effect sizes observed in this study may not be directly transferable to outpatient, day-clinic, or primary care settings, where treatment intensity, social context, and the natural course of both depression and loneliness may differ considerably. Similarly, the predominantly German-speaking sample and the use of German-language instruments limit the cultural generalizability of the findings, particularly given that the subjective experience and social determinants of loneliness are shaped by cultural norms regarding social contact and emotional disclosure. Nevertheless, the core research question — whether loneliness is associated with depression treatment outcomes — is relevant across healthcare systems, and the present study is intended to provide an empirical foundation that can be tested for replicability in other clinical populations and care settings. Therefore, the results may provide an evidence base and rationale for future clinical trials evaluating new treatment approaches in future intervention studies addressing loneliness in depression. Meta-analytic findings indicate that psychological interventions, especially cognitive-behavioral strategies targeting social cognition, can alleviate loneliness (99), underscoring their potential relevance for therapeutic approaches in depressive disorders. Furthermore, this study will contribute to a better understanding of the mechanisms linking loneliness and depression.

## 4 Ethics and dissemination

This study was approved by the Research Ethics Committee of Friedrich-Alexander-Universität Erlangen-Nürnberg (FAU) by vote on 19 November 2025 (No. 25-435-B). This study was developed in accordance with the Declaration of Helsinki. Informed consent is obtained from all of the individual participants included in this study. Any amendments affecting the study population, primary outcomes, or study procedures will be updated on ClinicalTrials.gov (NCT07333027) in a timely manner and reported transparently in future publications. Study findings will be submitted for publication in peer-reviewed journals and presented at national and international conferences. A summary of results will be provided to study participants upon request.
