# Correction: Clinical relevance of loneliness in the treatment of depression: study protocol for a naturalistic prospective longitudinal study

**DOI:** 10.3389/fpsyt.2026.2000383

**Published:** 2026-09-25

**Authors:** Franziska Sonnauer, Franca L. Fries, Maksym Druzenko, Ulrich W. Kastner, Christiane Mühle, Johannes Kornhuber

**Affiliations:** 1 Department of Psychiatry and Psychotherapy, Friedrich-Alexander-Universität Erlangen-Nürnberg (FAU), Universitätsklinikum Erlangen, Erlangen, Germany; 2 Department of Psychiatry, Addiction, Psychotherapy and Psychosomatic Medicine, Bezirkskliniken Mittelfranken, Erlangen, Germany

**Keywords:** biomarker, clinical sample, depression, loneliness, prognostic factor

In the published article, the ethical approval date was incorrectly given as 19 September 2025. A correction has been made to the section 4 **Ethics and dissemination,** Paragraph 1:

“This study was approved by the Research Ethics Committee of Friedrich-Alexander-Universität Erlangen-Nürnberg (FAU) by vote on 19 November 2025 (No. 25-435-B).”

The original version of this article has been updated.

