# Supplementary material for: Clinical relevance of loneliness in the treatment of depression: study protocol for a naturalistic prospective longitudinal study
Source: Front Psychiatry. 2026 Jul 24;17:1827062. doi: 10.3389/fpsyt.2026.1827062 (PMC13447386; doi:10.3389/fpsyt.2026.1827062)
Supplement: Supplementary file 1 [file SupplementaryFile1.docx]

Supplementary Material

[**1	Hypothesized biopsychosocial framework linking loneliness and depression	2**](#_Toc231799812)

[**2	Study flow chart	3**](#_Toc231799813)

[**3	Primary hypothesis	4**](#_Toc231799814)

[**4	Schematic formulation of the linear mixed model	5**](#_Toc231799815)

# 1 Hypothesized biopsychosocial framework linking loneliness and depression

*Figure S1* The figure depicts a hypothesized, literature-derived directional framework that motivates the study; it does not represent established effects. Loneliness, together with related psychosocial characteristics (reduced social network size, social isolation, low social support, limited social participation), is positioned as the upstream exposure (left). It is hypothesized to relate to clinical outcomes (right) — depressive symptom severity (MADRS, PHQ-9), treatment response and relapse, inpatient length of stay, quality of life, and suicidality — both directly and through intermediate mediating mechanisms (center): neuroendocrine (HPA axis), inflammatory, neurotrophic (BDNF), microbiota-gut-brain, and behavioral pathways. The arrow returning from depression to loneliness denotes the hypothesized reciprocal reinforcement, including reverse paths from depression to loneliness and its psychosocial correlates.

Arrows and colors: Solid arrows and solid (teal) boxes denote relationships central to the confirmatory study hypotheses — the association between loneliness and the course of depressive symptom severity, assessed with validated clinician-rated and self-report instruments at both recruitment sites. Dashed arrows and dashed (blue) boxes denote exploratory background mechanisms, examined only in the site-restricted biomarker/microbiome subsample and interpreted as hypothesis-generating. Behavioral and psychosocial variables additionally enter the primary model as covariates. Arrows represent hypothesized directional relationships derived from existing literature. Because the present study is non-interventional and observational, the planned analyses estimate associations consistent with this framework and cannot establish directionality or causation.

Abbreviations: BDNF – brain-derived neurotrophic factor; DJGLS – De Jong Gierveld Loneliness Scale; HPA – hypothalamic–pituitary–adrenal; MADRS – Montgomery–Åsberg Depression Rating Scale; PHQ-9 – 9-Item Patient Health Questionnaire; QoL – quality of life; SNI – Social Network Index; UCLA – University of California, Los Angeles Loneliness Scale.

# 2 Study flow chart

**Patients screened for eligibility across two study sites**

**Enrolled patients**

**Excluded**

- **not meeting inclusion criteria** (e.g. competing primary diagnosis other than depression [i.e., bipolar affective disorder, schizophrenia]; insufficient proficiency in the study language)
- **exclusion criteria** (e.g. pregnancy, earlier participation in this study)
- **declined participation / informed consent**
- **other**

**T0 Baseline, beginning of inpatient treatment**

- Clinical measures
- Biosamples^1^

**Inpatient treatment duration**

***t_0.1,_ t_0.2_ etc. to t_0.X_***

self-report questionnaire every 14 days

**T1 Discharge, end of inpatient treatment**

- Clinical measures
- Biosamples^1^

**T2 Follow-Up, 3 months after discharge**

- Clinical measures
- Biosamples^1^

***t_1.1,_ to t_1.5_***

self-report questionnaire every 14 days

- **Post-enrollment exclusion** (e.g., participants: treatment duration < 7 d)
- **Drop-out**

**3 months**

- **Drop-out**

*Figure S2* *Study Flow Chart.*

^1^ Footnote to Figure S2: Biosampling (blood, saliva, stool) is performed at the University Hospital site only.

# 3 Primary hypothesis

$$\mathbf{H0:}\beta1 \left( \mathrm{lon}_{T0_{c}} \right)=0 \wedge\beta2 \left( {\Delta lon}_{T0T1_{c}}:\mathrm{post}_{T0} \right) = 0 \wedge\beta3\left( \mathrm{lon}_{T0_{c}}:\Delta\mathrm{lon}_{T0T1_{c}}:\mathrm{post}_{T0} \right)=0$$

$$\mathbf{H1}\boldsymbol{:}\beta1\neq0 \vee\beta2\neq0 \vee\beta3\neq0$$

Abbrevations: H0 – null hypothesis; H1 – alternative hypothesis; β – regression coefficient; ∧ – logical AND; ∨ – logical OR; lon_T0_c – baseline loneliness measured with the 3-Item UCLA Loneliness Scale; ${\Delta lon}_{T0T1_{c}}$ – change in loneliness from T0 to T1, measured using the 3-Item UCLA Loneliness Scale at T0 and T1, assigned to subsequent measurements; $\mathrm{lon}_{T0_{c}}:\Delta\mathrm{lon}_{T0T1_{c}}:\mathrm{post}_{T0}$ – interaction between baseline loneliness and change in loneliness, assigned to subsequent measurements

Model assumptions: β₁ < β_3_ and β_2_ < β_3_; β_3_ = 2.0 MADRS points per 1 standard deviation increase in loneliness; β₁ = 1.75 MADRS points per 1 standard deviation increase in loneliness; β_2_ = 1.75 MADRS points per 1 standard deviation increase in loneliness

**T0 Loneliness**

**T0 Depression**

**T1 Loneliness**

**T1 Depression**

***Δ Loneliness (T0-T1)***

***Loneliness***

**T2 Loneliness**

**T2 Depression**

*Figure S3* *Associations tested as the primary hypothesis.*

# 4 Schematic formulation of the linear mixed model

$$\boldsymbol{MADRS}\boldsymbol{it}= \beta0 \left( intercept \right)+ \beta1 (association of baseline loneliness on MADRS at T0,T1, and T2) + \beta2 (change in loneliness from T0 to T1 on MADRS at T1 and T2) + \beta3 (interaction of baseline and change loneliness on MADRS at T1 and T2) + random slope (individual differences in MADRS across measurement points) + random intercept (individual differences in MADRS at baseline) + time effects covariates + further covariates + residual variance (\varepsilon)$$

The linear mixed model analysis examines depression symptom severity (MADRS), measured at T0, T1, and T2, as a function of loneliness (baseline, change in loneliness, baseline x change), time effects and further covariates. Model fit will be evaluated using omnibus likelihood ratio test. The following table S1 presents the time effects, background and content variables included in the primary analysis for this study. Further covariates (e.g. medication, social network, therapy participation and others) will be included in an exploratory approach.

Table S1. Variables of the primary linear mixed model

| **Variable label** | **Variable description** | **Scale level** | **Rationale for inclusion** |
| --- | --- | --- | --- |
| (zeit_num \| id) | Random Intercept + Random Slope [MADRS points; days] | continuous | depression symptom severity at T0 and change in depression symptom severity per day differs between persons |
| time effects | | | |
| dur_T0T1_c:at_T1 | inpatient treatment duration [days] | continuous | association of individual length of inpatient stay with MADRS at T1 |
| dur_T0T1_c:at_T2 | inpatient treatment duration [days] | continuous | association of individual length of inpatient stay with MADRS at T2 |
| lon_T0_c:at_T1 | measurement point-specific deviation of the baseline loneliness association at T1 relative to T0 [MADRS points per SD loneliness] | continuous | exploratory analysis of measurement points specific associations |
| lon_T0_c:at_T2 | measurement point-specific deviation of the baseline loneliness association at T2 relative to T0 [MADRS points per SD loneliness] | continuous | exploratory analysis of measurement points specific associations |
| $\Delta$lon_T0T1_c:at_T2 | measurement point specific deviation of the change in loneliness association at T2 relative to T1 [MADRS points per SD loneliness] | continuous | exploratory analysis of measurement points specific associations |
| background variables | | | |
| Age | Age [years of age] | continuous |  |
| Gender | Gender [male/female/other] | nominal (dummy-coded) |  |
| Site | Recruitment site [University hospital/community hospital] | nominal (dummy coded) |  |
| content variables | | | |
| Fsozu_T0  Fsozu_T1  Fsozu_T2 | Social Support [14-Item Social support measure] | continuous | established protective and potential modulating factor between depression and loneliness |
| Spin_T0  Spin_T1  Spin_T2 | Social Anxiety [17-Item Social anxiety measure] | continuous | prevalent comorbidity in clinical populations, important to distinguish the associations between social anxiety, depression and loneliness |
| further explorative covariates | | | |
